# Recent Advances in Silicon Quantum Dot-Based Fluorescent Biosensors

**DOI:** 10.3390/bios13030311

**Published:** 2023-02-23

**Authors:** Yanan Zhang, Ning Cai, Vincent Chan

**Affiliations:** 1 College of Chemistry and Chemical Engineering, Huanggang Normal University, Huanggang 438000, China; 2 Key Laboratory for Green Chemical Process of Ministry of Education, Hubei Engineering Research Center for Advanced Fine Chemicals, Hubei Key Laboratory of Novel Reactor & Green Chemical Technology, School of Chemical Engineering and Pharmacy, Wuhan Institute of Technology, Wuhan 430073, China; 3 Department of Biomedical Engineering, Khalifa University of Science and Technology, Abu Dhabi 127788, United Arab Emirates

**Keywords:** silicon quantum dot, biosensors, photoluminescent, fluorescence detection, bioimaging

## Abstract

With the development of nanotechnology, fluorescent silicon nanomaterials have been synthesized and applied in various areas. Among them, silicon quantum dots (SiQDs) are a new class of zero-dimensional nanomaterials with outstanding optical properties, benign biocompatibility, and ultra-small size. In recent years, SiQDs have been gradually utilized for constructing high-performance fluorescent sensors for chemical or biological analytes. Herein, we focus on reviewing recent advances in SiQD-based fluorescent biosensors from a broad perspective and discussing possible future trends. First, the representative progress for synthesizing water-soluble SiQDs in the past decade is systematically summarized. Then, the latest achievement of the design and fabrication of SiQD-based fluorescent biosensors is introduced, with a particular focus on analyte-induced photoluminescence (fluorescence) changes, hybrids of SiQDs with other materials or molecules, and biological ligand-modification methods. Finally, the current challenges and prospects of this field are highlighted.

## 1. Introduction

Silicon is the second most abundant element in the Earth’s crust. In 1823, Berzelius obtained it in a pure form for the first time. With the advances of nanotechnology, silicon nanomaterials have been applied in biology, chemistry, medicine and other fields [1,2,3]. Among them, photoluminescent (fluorescent) silicon nanomaterials exhibit good optical stability and degradability in physiological environments [4,5,6]. More recently, silicon quantum dots (SiQDs), which are zero-dimensional fluorescent silicon nanomaterials with lower toxicity than II-VI QDs (e.g., CdSe) and III-V QDs (e.g., InP), have emerged as promising photoluminescent biosensors. In 1992, Littau et al. first reported SiQD crystals [7]. Because of their attractive characteristics, such as good biocompatibility, excellent optical properties and controllable surface functionalization [8,9], SiQDs are very likely to replace traditional QDs containing heavy metals, endowing the field with a promising “green” photoluminescent material.

As an indirect-band gap semiconductor, bulk silicon shows poor photoluminescent properties due to the low probability of electron-hole pair recombination, which limits its optical applications. Compared with bulk silicon, SiQDs possess special optical properties attributed to quantum confinement, as well as size and surface effects. When the radius of a silicon material is smaller than the exciton Bohr radius of silicon (~4.2 nm), the quantum confinement effect will occur [5,10]. The SiQDs in this state have an optically physical characteristic similar to that of a direct bandgap semiconductor. For cubic SiQDs at a three-dimensional scale (*x*, *y*, *z*), the energy difference between electrons and holes can be approximated as the sum of the band gap energy of silicon (*E_g_*) and the energy contributions from the conduction and valence bands across the entire space of the confined cubical dimensions, as follows [11,12]:

$$\Delta E=E_{g}+\underset{i=x,y,z}{\sum }\frac{\hbar^{2}\pi^{2}n_{i}^{2}}{2d_{i}^{2}}\left(\frac{1}{m_{e}}+\frac{1}{m_{h}}\right)$$

where *d_i_* is the length of the cubic SiQDs in the *i*th dimension, *n* is the quantum number, and *m_e_* is the effective mass of the conduction band electrons and *m_h_* the effective mass of the valence band holes. This confinement effect (when *d_i_* approaches the Bohr radius) causes the energy gap of silicon to widen, enhancing the radiation recombination of holes and electrons. Thus, SiQDs become effective fluorophores following the enhancement of their luminescence efficiency against the increase in confinement energy, as mentioned above [13]. At the same time, the class of SiQDs reviewed herein are readily excited by a Xe lamp (200 to 800 nm) or specific lasers for the real-time detection of fluorescence (photoluminescence) intensity by a conventional fluorescence spectrometer or fluorescence microscope according to the following linear relationship [14]:

$$F=2.303\varphi I_{0}\varepsilon Cx$$

where *φ* is the fluorescence quantum yield, *I*_0_ is the intensity of incident light for excitation, *ε* is the molar extinction coefficient, *C* is the concentration of the fluorescent substance, and *x* is the optical path of the excitation light. Furthermore, with a reduction in size, the photoluminescence of SiQDs can be blue-shifted [15,16,17]. The larger-size silicon nanocrystals are more stable due to their lower surface-to-volume ratio [18]. On the other hand, the surface groups of SiQDs also influence their photoluminescence properties [10].

At present, visible-to-near-infrared (NIR) photoluminescent SiQDs have been reported [16], but most of them still have some problems. For example, the surface of SiQDs synthesized by the typical “top-down” hydrofluoric acid (HF) etching method is mostly abundant in Si–H bonds, which are easily oxidized and highly hydrophobic. Therefore, hydrophilic molecules such as octadecenoic acid and allylamine are deposited on the surface of the SiQDs for enabling their application in biological analysis [19,20]. In this way, it requires at least two steps to obtain water-soluble SiQDs, which is a tedious process. At the same time, surface modification can change the physical or chemical properties of SiQDs, such as increasing size and decreasing quantum yield (QY), which is not conducive to cell entry and biological imaging. The QY of most SiQDs is in the range of 15–32.8%. When applied to cells, tissues or in vivo imaging, they are susceptible to interference from background photoluminescence. Then, the sensitivity of the detection methods is affected, especially for the blue and green photoluminescent SiQDs [2,5,21]. Studies have shown that NIR (650–900 nm) photoluminescent nanoparticles possess the advantages of little interference from typical background of biological samples, weak light scattering, and almost undetectable damage to living organisms. They display unique merits in cell and in vivo imaging [22,23]. The development of NIR SiQDs has become one of the hot research directions. Veinot’s group prepared SiQDs with an Si–H bond surface by high-temperature decomposition (>1000 °C) and HF etching. They further treated the SiQDs with Br_2_ to make a Si–Br bond surface. Finally, NIR SiQDs (~3.4 nm) with alkyl end were prepared via the reaction between the Si–Br group and the format reagent [10]. Ozin’s group achieved cellular imaging by improving the water solubility of NIR SiQDs (~4.4 nm) via modification with polyethylene glycol [23]. However, the current preparation conditions for NIR SiQDs are usually harsh and most of the obtained NIR SiQDs are hydrophobic.

Note that the water solubility of SiQDs is critically significant for biological applications. In the past decade, researchers have employed various methods to synthesize water-soluble SiQDs [24,25,26,27]. SiQDs mainly comprise silicon nanodots (SiNDs) and photoluminescent silicon nanoparticles (SiNPs), which both referred as photoluminescent silicon nanocrystals. According to the categorization of nanoparticles by Park et al. [28], we use SiQDs to represent this class of nanomaterial. The optical applications of SiQDs are focused on photoluminescent imaging [5,9]. Recently, SiQDs have been gradually deployed for the fabrication of biosensors. For instance, SiQD-based ratiometric photoluminescent biosensors integrated the response and reference signals, thereby improving their detection accuracy [29]. In comparison with organic (fluorescent) dyes, SiQDs demonstrate higher quantum yields in an NIR regime, higher emission lifetimes (over 5 ns), higher photophysical stability towards NIR excitation, a broader spectrum in the absorption of incident radiation, and convenience for tuning excitation/emission wavelength [30]. Recently, SiQD-based photoluminescent sensors have been successfully applied to probe pH, ionic species, DNA, proteins, dopamine, etc. [31]. Currently, several types of photoluminescent biosensors have been developed for detecting environmental and physiological markers such as Cu^2+^/F^−^ [32], Cd^2+^ [33], Fe^3+^ [34,35] and DNA [36]. In this review, we summarize the recent advances in the direct preparation of SiQDs with good water solubility and their applications in biosensors. First, we introduce the strategies for synthesizing SiQDs and focus in representative “bottom-up” synthesis approaches. Based on the great progress in the synthesis of water-soluble SiQDs, the related works about SiQD-based biosensors are further reviewed. Finally, some perspectives and outlooks concerning the synthesis and bioapplications of SiQDs are discussed.

## 2. Direct Synthesis of Water-Soluble SiQDs

The luminescence properties and water solubility of SiQDs are closely related to the structure of precursors, synthesis routes, size and surface groups. To date, the methods for the synthesis of SiQDs can be mainly divided into two categories: top-down and bottom-up approaches [5]. Extensive efforts are dedicated to developing effective strategies for preparing hydrogen- or halogen-terminated SiQDs by the “top-down” methods, which still need further surface modification in order to enhance their solubility and stability for broad application [26]. Notably, recent years have witnessed the growth of various “bottom-up” techniques for the direct synthesis of SiQDs in aqueous solutions, facilitating this promising nanomaterial for bioapplication [18,21,25]. The synthetic approaches include microwave and UV irradiation, hydrothermal, and stirring methods. Table 1 displays the photoluminescent properties of SiQDs synthesized by these methods. As shown above, N-containing silicon sources are widely used for the preparation of SiQDs, which may result in most SiQDs emitting blue or green photoluminescence. Generally, SiQDs are spherical particles with a diameter of approximately 2–5 nm per transmission electron microscopy (TEM) characterization. Recent developments of water-soluble SiQDs obtained by the “bottom-up” methods are reviewed in this section.

### 2.1. Microwave or UV Irradiation Method

**Figure 1:**
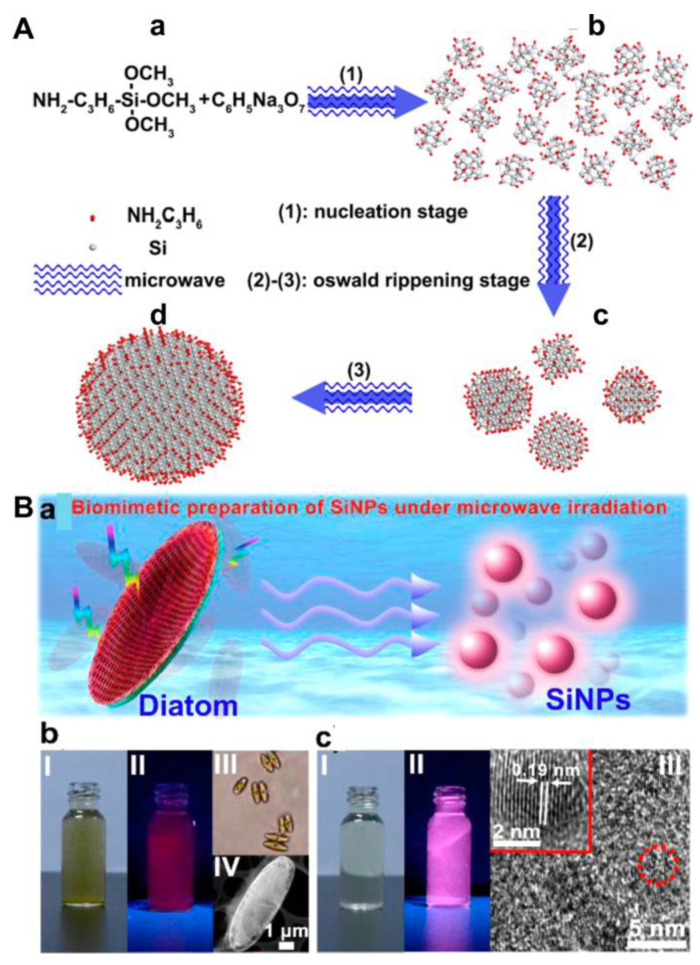
(**A**) Schematic illustration of bottom-up one-pot synthesis of SiQDs. (a) reaction precursor; (b) twenty-one nuclei; (c) four small-size nanocrystals; (d) one large-size nanocrystal. Reproduced from [18], with permission from American Chemical Society, 2013. (**B**) (a) Schematic illustration of biomimetic synthesis of SiQDs. (b) I and II indicate the diatom precursor solution under ambient light (left) and 365 nm irradiation (right), respectively; III and IV present a microscope photo and TEM image of the diatoms, respectively. (c) I and II indicate the as-prepared SiQD sample solution under ambient light (left) and 365 nm irradiation (right), respectively; III shows an high resolution TEM (HRTEM) image of the SiQDs with high crystallinity. The inset in (III) presents the enlarged HRTEM image of a single SiQD. Reproduced from [37], with permission from American Chemical Society, 2015.

To date, the microwave irradiation (MWI) methodology has been gradually employed for synthesizing various types of silicon nanomaterials, such as nanodots, nanorods and nano-shuttles [39,55,56]. This method possesses three dominant merits in comparison with conventional heating strategies. First, a high reaction rate can be achieved as the sample temperature is rapidly raised. Second, reaction selectivity can be improved under MWI because various substances have different dipole constants. Finally, a rather uniform structure can be formed due to the homogeneous heating induced by microwaves. The most significant potential benefit of the MWI-enabled synthesis of quantum nanodots is the enhancement of photoluminescence intensity without shifting the absorbance/emission spectrum [57]. In 2013, using the MWI method, Zhong et al. adopted APTMS and trisodium citrate (TC) as precursors to synthesize highly photostable SiQDs in aqueous solutions [18]. In brief, the SiQDs were obtained through a nucleation stage and an Ostwald ripening stage (Figure 1A). APTMS (C_6_H_17_NO_3_Si) molecules were reduced by TC (C_6_H_5_Na_3_O_7_) under MWI, generating crystal nuclei in the first step (step (1)). When the concentration of APTMS molecules decreased to certain degree, the nucleation stage stopped, and the Ostwald ripening stage began (step (2)) and continued (step (3)). Eventually, large-size silicon nanocrystals were formed by the dissolution and absorption of unstable small nanocrystals. Since then, water-soluble SiQDs with good size distribution (2.2–4.22 nm) have been easily synthesized within 25 min via the MWI method (Table 1). In addition, most SiQDs are prepared below 200 °C. Without the deployment of chemical reagents, Wu et al. presented a new biomimetic strategy for the synthesis of SiQDs with a longer emission wavelength (620 nm) under MWI (Figure 1C) [37]. Zhong et al. further applied the ultraviolet (UV) irradiation method to synthesize tunable-color SiQDs in an aqueous phase with a similar process as the MWI method (Figure 1B) [40]. It is noted that the synthesis was performed in glass flasks at room temperature and atmospheric pressure. The quantum yield (QY) and size of SiQDs obtained by the two irradiation methods were alike by using the same silicon source [18,40]. As the QY of SiQDs is in the range of 0.3–47% by the UV and MWI methods, there is still room for improvement in achieving high QY SiQDs. On the other hand, although the two methods are facile and rapid, they have not been widely employed in SiQD preparation. Finally, the applications of these SiQDs are focused on the photoluminescent imaging of cancer cells, C. elegans and zebrafish [18,37,39,40], while there is relatively little work on their applications in biosensing. On the other hand, MWI-enabled synthesis may induce cytotoxicity in the resulting quantum dots [58].

### 2.2. Hydrothermal Method

**Figure 2:**
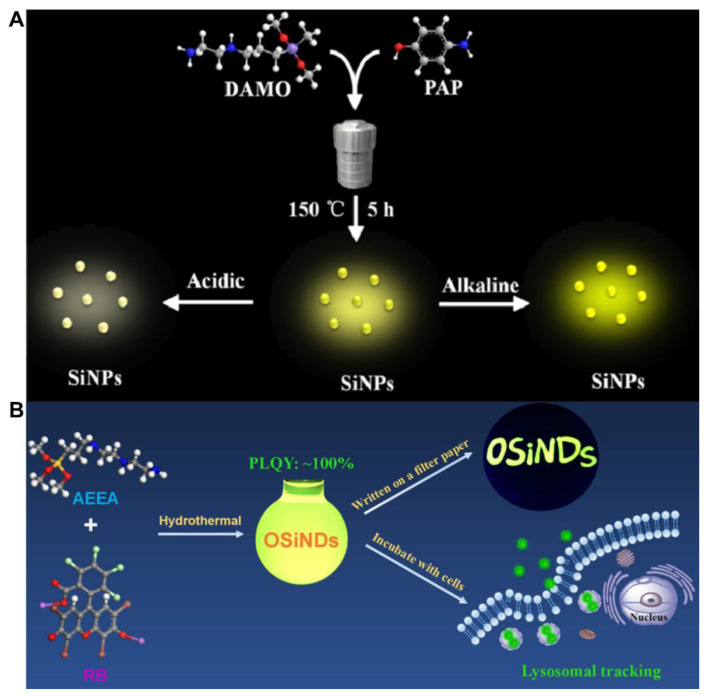
(**A**) Schematic illustration of the one-pot synthesis of yellow-green-emissive SiNPs and SiNPs for pH detection. Reproduced from [27], with permission from American Chemical Society, 2021. (**B**) Schematic illustrating the synthesis of green-emitting organosilica nanodots (OSiNDs) with ultrahigh quantum yields and their application in long-time lysosomal imaging. Reproduced from [48], with permission from American Chemical Society, 2018.

The hydrothermal method refers to a method in which the reaction system is heated to a critical temperature or close to that level in a sealed container with specific solvents as the medium, to generate high pressure and realize inorganic synthesis or material preparation [59]. In general, the hydrothermal method of quantum nanodot synthesis offers the unique advantage of low setup costs, better environmental sustainability and a product with a higher quantum yield [60]. At present, this method has become popular for preparing water-soluble SiQDs. As shown in Table 1, blue to yellow-green emitting SiQDs were facilely obtained by the one-pot hydrothermal method with a temperature range of 150–200 °C. For instance, Na et al. employed DAMO and 4-aminophenol as the reactants for the hydrothermal synthesis of pH-sensitive SiQDs (Figure 2A). The developed sensor can be used to monitor the pH changes in live HepG2 cells [27]. The QY of SiQDs obtained by this method is in the range of 1.6–100% (Table 1). High QY SiQDs can effectively resist the interference of background photoluminescence. Chen et al. synthesized ultrabright water-soluble SiQDs (100% QY) using AEEA and rose bengal (RB) via hydrothermal treatment for 4 h (Figure 2B). The SiQDs were applied to long-time lysosomal imaging in A549 lung cancer cells [48]. The reaction time (2–20 h) of the method is much longer than that of the microwave and UV irradiation methods (within 30 min) (Table 1). Other potential shortcomings of the hydrothermal method for the synthesis of SiQDs include low product yield, low quantum yield and lower stability [61]. Moreover, researchers are prone to using this method for preparing N-doped SiQDs that usually emit blue-to-yellow-green photoluminescence. In 2022, Wei et al. utilized MPTMS as a source of silicon for the synthesis of sulfhydryl-functionalized SiQDs (S-SiQDs) by the hydrothermal method [50]. While the m-phenylenediamine (m-PD) reagent was also used as a precursor that introduced the N element to the final product, the maximum emission of the S-SiQDs (492 nm) was still in the short wavelength region [50].

### 2.3. Stirring Method

**Figure 3:**
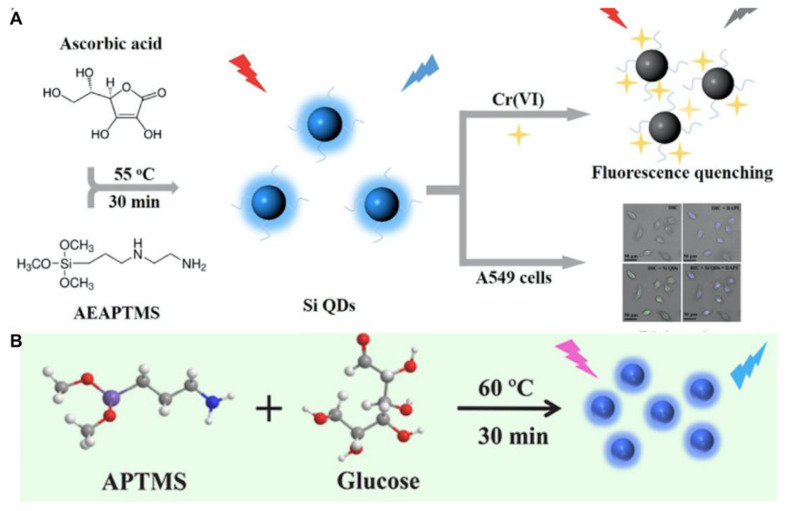
(**A**) Schematic illustration of the detection of Cr(VI) and bioimaging using SiQDs as a photoluminescent probe. Reproduced from [52], with permission from Elsevier, 2018. (**B**) Schematic illustration of the SiQD synthesis process. Reproduced from [54], with permission from Elsevier, 2022.

The stirring method is generally carried out in a glass flask at room pressure. For the preparation of SiQDs, this method demonstrates advantages such as relatively simple operation, mild reaction conditions, rapid process and low cytotoxicity of produced nanodots compared tot the two methods mentioned above [62]. Since ascorbate sodium (AS) possesses a higher reduction property than trisodium citrate, Wang et al. adopted AS and APTES to facilely synthesize green-emitting SiQDs under stirring for 30 min at room temperature [25]. The photoluminescent lifetime of the SiQDs was as long as 10.2 ns, which is beyond that of the auto-photoluminescence of cells (~2 ns) and native fluorophores (<5 ns). The SiQDs could be well-qualified for long-term cell imaging [25]. By the stirring method, Phan et al. used DAMO and L-ascorbic acid (AA) to prepare excitation-dependent SiQDs at 55 °C. They further applied the SiQDs to the detection of Cr(VI) and the photoluminescent imaging of A549 cells (Figure 3A). They found that the dilution of SiQDs with water could lead the emission peak of SiQDs to blue-shift [52]. The volume of water may influence the energy-transfer process and surface ligands of SiQDs [63,64,65]. The initial mass of precursors and reaction volume may also affect the intrinsic luminescence of silicon nanocrystals. Wang’s and Su’s groups both employed APTMS and glucose to synthesize SiQDs under stirring for 30 min at 60 °C (Figure 3B). Since the mass of reactants and solvent volume are not the same, the size, QY and photoluminescent properties of the two SiQDs are quite different [53,54]. However, the disadvantage of the stirring method for nanodot production is the formation of large aggregates [66].

## 3. Design and Fabrication of SiQD-Based Photoluminescent Biosensors

Attributed to the elegant work of the direct preparation of water-soluble SiQDs, various kinds of SiQD-based photoluminescent biosensors have been elaborately developed. The target analytes include lactate dehydrogenase, hydrogen ion, glucose, heavy metal ions, DNA, thiols, cancer cells and bacteria [41,42,43,67,68,69,70,71,72]. The sensing mechanisms of the SiQD-based biosensors for the analytes are based on metal-enhanced fluorescence (MEF) [46], inner filter effect (IFE) [68], fluorescence resonance energy transfer (FRET) [73], surface energy transfer (SET) [74] and so forth. Various design tactics have been exploited for the fabrication of these biosensors. Herein, we summarized that the strategies were focused on analyte-induced photoluminescent changes, hybrids of SiQD with other materials or molecules, and biological ligand-modification methods. Table 2 summarizes the performance of SiQD-based biosensors for the detection of various analytes:

### 3.1. Analyte-Induced Fluorescence Changes

**Figure 4:**
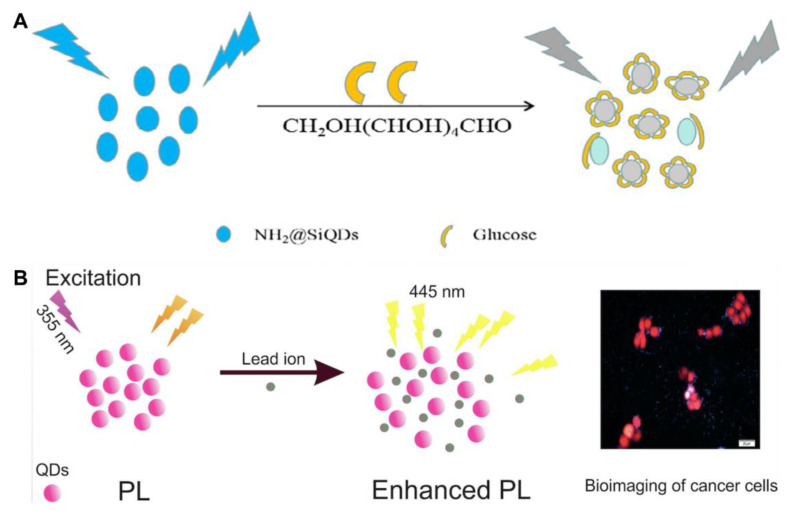
(**A**) Schematic illustration of the free-enzyme biosensor for sensitive glucose detection. Reproduced from [67], with permission from Elsevier, 2019. (**B**) Schematic illustration of the nanosensor developed for lead detection. Reproduced from [46], with permission from Elsevier, 2021.

The photoluminescence of SiQDs synthesized via different methods can be directly quenched or enhanced by certain molecules or ions, and turn-off/on biosensors have been developed for the detection of target analytes (e.g., pH [27], Hg^2+^ [42], CrO_7_^2−^ [45], glucose [67]). Du et al. prepared amino-functionalized SiQDs (NH_2_@SiQDs) via the microemulsion method by using silicon tetrachloride and allylamine as the respective silicon source and functional group [67]. A non-enzymatic glucose sensor was designed on the principle of the photoluminescent quenching of glucose towards NH_2_@SiQDs (Figure 4A). The limit of detection (LOD) was determined to be 0.3 μM. The linear response range against glucose was 1–90 μM, which did not cover the glucose level (3.9–6.1 mM) in the blood of a healthy human [76]. Finally, the sensor was used for the determination of glucose in 1% human serum [67]. Shen et al. also synthesized N-doped SiQDs using APTMS, trisodium citrate and urea by the hydrothermal method [42]. The photoluminescence of the N-SiQDs could be rapidly quenched within 30 s in the presence of Hg^2+^ via static quenching. After the addition of glutathione (GSH) to the Hg^2+^–N-SiQD system, the photoluminescence was restored within 1 min. This is because GSH competes with N-SiQDs for the capture of Hg^2+^. The detection limits for Hg^2+^ and GSH are 24 nM and 55 nM, respectively. The “on-off-on” photoluminescent sensor has been applied to the dual-responsive detection of Hg^2+^ and biothiols in live MCF-7 cells [42]. MEF occurs when the separation between the fluorophore and metallic surface is about 5–90 nm, facilitating more photons being absorbed by the fluorophore [46]. Golsanamlou et al. prepared amine-functionalized SiQDs for the turn-on detection of Pb^2+^ based on the MEF effect (Figure 4B). The photoluminescence of the SiQDs increased linearly with the Pb^2+^ concentration with an LOD down to around 20 ng/mL. The developed nanosensor was utilized for Pb^2+^ detection in plasma, cell lysate and HT 29 cancer live cells. The synthesis of SiQDs via hydrothermal technique is simple but time-consuming (20 h) [46]. Wei et al. found that the photoluminescence of S-SiQDs was selectively quenched by hypochlorite (ClO^−^) via the oxidization of the sulfhydryl groups of S-SiQDs and static quenching [50]. The reaction could be completed in 10 s. The photoluminescent intensity of S-SiQDs in response to ClO^−^ was in a linear range of 0.05–1.8 μM with a detection limit of 13 nM. The S-SiQDs were successfully applied to the imaging of ClO^−^ in MCF-7 cells and zebrafish [50].

### 3.2. Hybrid with Other Materials or Molecules

**Figure 5:**
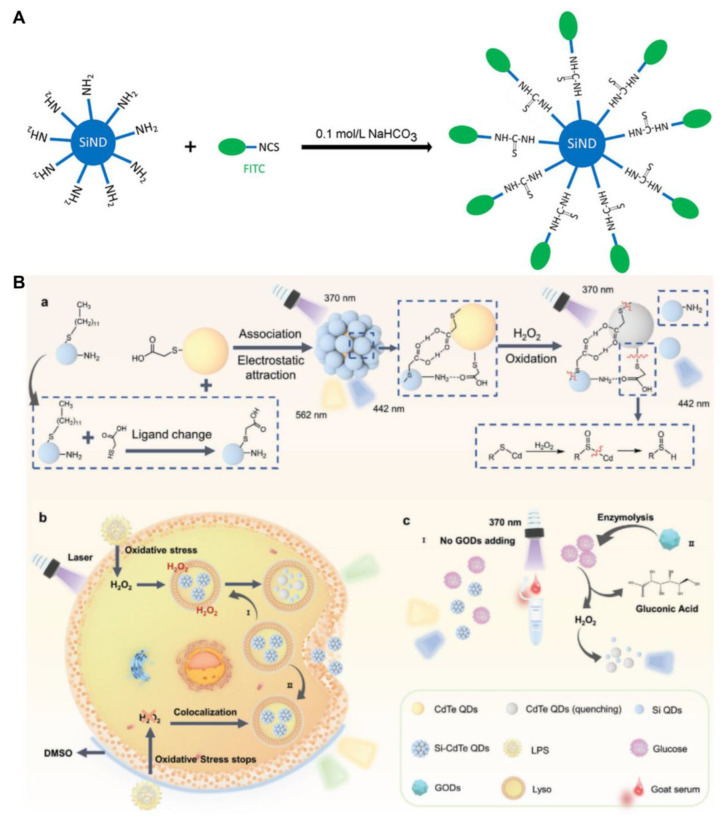
(**A**) Schematic of the ratiometric pH sensor: FITC-modified SiQD. Reproduced from [69], with permission from Elsevier, 2020. (**B**) Schematic diagram of (a) the synthesis of a Si-CdTe QDs probe for the detection of (b) H_2_O_2_ in cells and (c) glucose in goat serum, respectively. Reproduced from [73], with permission from Willey, 2022.

For this kind of SiQD-based biosensor, the general design tactic is to hybridize SiQDs with other materials or molecules, producing signal changes in the presence of targets [77]. Our group developed a ratiometric DNA sensor by simply mixing SiQDs and Ru(bpy)_2_(dppx)^2+^ [68]. Ru(bpy)_2_(dppx)^2+^ is a molecular “light switch” complex that can selectively recognize double-stranded DNA. The IFE of Ru(bpy)_2_(dppx)^2+^ toward SiQDs is not affected by its intercalation into DNA. The ratiometric photoluminescent detection of DNA was achieved by using SiQDs (448 nm) and Ru(bpy)_2_(dppx)^2+^ (601 nm) as the reference and response signals, respectively. In aqueous solutions, the I_601_/I_448_ intensity ratio of SiQD–Ru(bpy)_2_(dppx)^2+^ increased linearly with DNA concentration in a range of 20–1500 nM. The ratiometric sensor could be used for the visual detection of DNA at the nanomolar level. The LOD was as low as 4.3 nM. Moreover, the established method was suitable for the photoluminescent detection of the target in 1% human serum [68]. Though hydrogen bonding may exist between the amino groups on the surface of SiQDs and the dppx ligand, this type of sensor is not appropriate for the cellular environment. The main reason is probably the uneven distribution ratio of SiQDs and Ru(bpy)_2_(dppx)^2+^ in living cells. We also developed a ratiometric pH sensor via a simple covalent method employing amino-terminated SiQDs and fluorescein isothiocyanate (FITC) (Figure 5A). The photoluminescent sensor was facilely fabricated by the modification of SiQDs with FITC under alkaline conditions. The SiQD in SiQD-FITC serves as a nanocarrier and a reference. Meanwhile, the FITC in SiQD-FITC preserves its function for pH sensing. The SiQD-FITC sensor was successfully applied to the ratiometric imaging of pH in live MCF-7 cells [69].

Feng and colleagues used SiQDs and CdTe QDs to prepare dual-emission Si-CdTe QDs for the ratiometric detection of H_2_O_2_ and glucose through the FRET mechanism (Figure 5B) [73]. The Si-CdTe QDs were formed via three bonding forces. First, free thioglycolic acid (TGA) in CdTe QDs could replace 1-dodecanethiol on the surface of SiQDs by nucleophilic substitution, generating TGA-capped SiQDs. Then, the SiQDs and CdTe QDs formed an association by their carboxylic acids in TGA caps. Second, the amino groups of SiQDs reacted with the carboxyl groups of CdTe QDs to form ammonium salts. Third, electrostatic adsorption occurred between SiQDs and CdTe QDs with opposite electric charges. The TGA on the surface of Si-CdTe QDs could be oxidized in the presence of H_2_O_2_. Then, the FRET between SiQDs and CdTe QDs was interrupted, causing photoluminescent changes. The Si-CdTe QDs exhibited low cytotoxicity stemming from SiQDs, which are fit for photoluminescent imaging of H_2_O_2_ in live HeLa cells. Furthermore, with the help of glucose oxidase (GOD), D-glucose can be oxidized to release D-gluconic acid and H_2_O_2_. The detection limits for H_2_O_2_ and glucose are 79 and 140 nM, respectively. The developed sensor has also been used for ratiometric detection of glucose in goat serum [73]. A photoluminescent GSH sensor based on a MnO_2_–SiQDs nanocomposite has been developed by Ma et al. [74]. The SiQDs adhere to the surface of the MnO_2_ nanosheet dominated by electrostatic interactions. The SET from SiQDs to MnO_2_ induces the photoluminescence of SiQDs to decrease. The MnO_2_ nanosheets are reduced in the presence of GSH, and the photoluminescence of SiQDs can be restored. The sensor exhibits a detection limit of 153 nM for GSH. It can be applied to directly sense GSH in live BHK cells [74].

### 3.3. Biological Ligands-Modification

**Figure 6:**
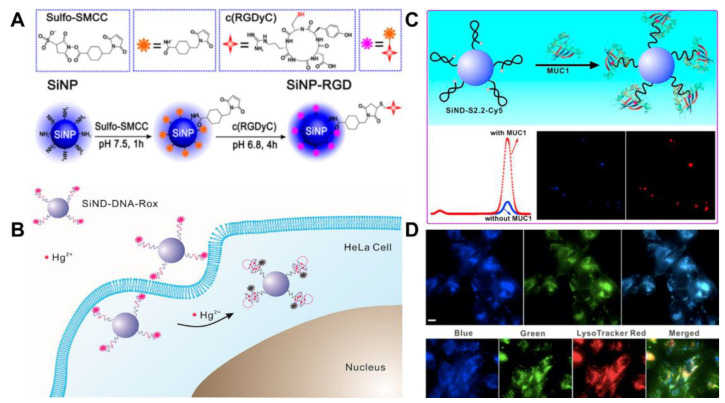
(**A**) Schematic synthesis of the SiNPs-RGD. Reproduced from [79], with permission from American Chemical Society, 2015. (**B**) Schematic presentation of the SiND-DNA-Rox sensor for the ratiometric detection of Hg^2+^ in HeLa cell. Reproduced from [70], with permission from American Chemical Society, 2018. (**C**) Schematic presentation of the SiND-S2.2-Cy5 aptasensor for the detection and bioimaging of MUC1. Reproduced from [75], with permission from Elsevier, 2018. (**D**) photoluminescent images of MCF-7 cells (Top row) incubated with SiNDs-Apt-FAM (from left to right: blue channel, green channel, merged), and photoluminescent images of MCF-7 cells (bottom row) incubated with SiNDs-Apt-FAM and LysoTracker Red DND-99 (from left to right: blue channel, green channel, red channel, merged). Scale bars: 20 µm. Reproduced from [71], with permission from Elsevier, 2017.

The biomolecular ligands used for the construction of SiQD-based biosensors mainly include DNA [70,71], antibody [78] and peptide [79]. Yanagawa et al. synthesized water-dispersible NIR SiQDs with amino and epoxy groups functionalized on the surface via high-temperature treatment (1100 °C), HF etching and surface functionalization [78]. IgG antibodies are bound covalently to the SiQDs. The luminescence property of the SiQDs is not influenced by the conjugation. Meanwhile, IgG antibodies in IgG-SiQDs retain the binding property with antigen nucleoprotein [78]. The team used a peptide containing arginine–glycine–aspartic acid sequence (c(RGDyC)) to conjugate with amino-terminated SiQDs via a crosslinker 4-(N-maleimidomethyl)cyclohexane-1-carboxylic acid 3-sulfo-N-hydroxysuccinimide ester sodium salt (Sulf-SMCC) (Figure 6A). The SiQDs-RGD exhibited cell specificity to integrin α_v_β_3_-positive U87MG over integrin α_v_β_3_-negative MCF-7 cells. Moreover, the biological sensor possesses efficacious destructiveness to the U87MG cells, suggesting the probability of simultaneous cancer detection and treatment [79].

Compared with other ligands, DNA has many advantages [80,81]: (1) DNA possesses high stability and reversibility. (2) Biosensors can be designed based on DNA secondary structures (e.g., double-stranded, four-stranded and hairpin structures), which can be predicted more easily than secondary structures from antibody or peptide sequences. (3) The binding of DNA aptamers to the target is usually accompanied by structural changes, which facilitates the design of sensors. (4) DNA can be screened in vitro, and the cost of synthesis is relatively low. (5) The sequence of DNA can be combined, optimized and easily modified, which promotes its application in the field of nanotechnology. Because DNA aptamers are selected in vitro through the evolutionary process of exponential enrichment, they have strong specificity and high affinity for the target [81]. In our previous works, a series of aptamer-functionalized SiQDs were prepared for the photoluminescent detection of various analytes. For example, a ratiometric photoluminescent Hg^2+^ sensor was developed by the covalent attachment of SiQDs to the Hg^2+^-specific 6-carboxy-X-rhodamine (Rox)-tagged DNA (Figure 6B). Owing to DNA structure change, the signal change of the constructed photoluminescent sensor was only related to the target ion based on the T-Hg^2+^-T interaction. Dual-color analysis of intracellular Hg^2+^ was realized by photoluminescent imaging and flow cytometry [70]. A photoluminescent SiQD-based aptasensor for the ratiometric detection of biomarker mucin 1 (MUC1) was also constructed by the covalent coupling method using the crosslinker Sulf-SMCC. For the sensor, the photoluminescence of cyanine (Cy5)-tagged aptamer S2.2 could be quenched by the SiQD nanocarrier in the absence of MUC1 but restored with the addition of MUC1 attributed to the structure switching of S2.2 (Figure 6C). The aptasensor exhibits a low detection limit (1.52 nM) for MUC1, which has been applied to the quantitative determination of the target in human serum and the distinction of MUC1 overexpressed cancer cells from normal cells [75]. Further study on the application of SiQDs in cell organelles was carried out. Using the characteristics of the ultra-small size of SiQDs and the specificity of aptamer AS1411 to nucleolin, a ratiometric photoluminescent aptasensor for the measurement of pH in lysosomes was constructed [71]. The pH-sensitive fluorescein tagged AS1411 (Apt-FAM) was employed for the covalent modification of SiQDs via the Sulf-SMCC. The developed aptasensor can recognize cancer cells and concentrate in the lysosomes of MCF-7 cancer cells (Figure 6D). We utilized the ratiometric sensors as mentioned above to gradually realize dual-color cellular imaging, cell-targeted recognition, and lysosomal imaging analysis. By a non-coupling method, a Cy5-DNA/SiQD-based biosensor was developed for the quantitative detection of nucleic acid in human fluid samples [82]. Since there is only an electrostatic interaction between Cy5-DNA and SiQDs, the distribution of Cy5-DNA and SiQDs may be uneven in live cells. The photoluminescent signal changes cannot be accurately determined, and the sensor was not fit for cellular imaging analysis.

## 4. Summary and Perspectives

This review has summarized the recent advances in the synthesis of water-soluble SiQDs and bioanalysis based on SiQDs for biosensor application through the photoluminescent detection of various ions and biomarkers. Since the first-deployed MWI method for the direct synthesis of water-soluble SiQDs was developed in 2013, many new strategies have been proposed for preparing SiQDs with good water dispersity and enhanced QY, simplifying the synthetic scheme and obtaining less hazardous chemicals since then. At the same time, NIR SiQDs were synthesized and modified with hydrophilic molecules for biosensing application. Owing to the great advancement in the preparation of water-dispersible SiQDs, they have emerged as novel photoluminescent probes for biosensor construction. As a result, several SiQD-based biosensors were designed for the sensing of various molecular targets in vitro or in vivo. In addition, several photoluminescent sensors based on SiQDs were constructed to improve bioanalytical performance, such as SiQDs-based ratiometric biosensors and DNA covalently functionalized SiQDs.

However, there are still several challenges that need to be overcome. First, to reduce the interference of background photoluminescence, the luminescence properties of SiQDs are mainly regulated from two aspects: increasing QY and adjusting emission wavelength towards the NIR region. As such, a more simple, rapid and green method for preparing water-soluble NIR SiQDs with high QY should be explored to facilitate in vivo bioapplications. Then, DNA signal amplification techniques can be introduced into the SiQD-based biosensor construction to achieve ultrasensitive detection, such as rolling circle amplification, hybrid chain reaction and catalytic hairpin assembly. Since the particle size of SiQDs is usually no more than 5 nm, and the length of ten DNA base sequences is about 3.3 nm [80], the steric hindrance effect should be balanced by the repulsive force between DNA strands in designing aptamer-functionalized SiQDs biosensors. The valence of aptamer-functionalized SiQDs needs to be more precisely controlled. Finally, the large-scale preparation and deeper bioapplications (e.g., real-time monitoring of drug release, gene therapy, and cancer therapy) of water-soluble SiQDs should be explored.

## Figures and Tables

**Table 1:** Some properties of water-souble SiQDs synthesized by the “bottom-up” methods.

Precursors	Methods	T (°C)	Time	Size (nm)	QY (%)	Ex (nm)	Em (nm)	FL (ns)	Ref.
APTMS/TC Diatom DAMO/L-glutathione APTES/TC APTMS/1,8-naphthalimide APTMS/TC APTMS/TC APTMS/TC/urea APTMS/TEPA DAMO/m-PD DAMO/4-aminophenol APTES/quercetin	Microwave Microwave Microwave Microwave UV irradiation Hydrothermal Hydrothermal Hydrothermal Hydrothermal Hydrothermal Hydrothermal Hydrothermal	160 150 — 180 20–25 200 180 200 200 180 150 200	10 min 10 min 8 min 25 min 30 min 2 h 5 h 4 h 12 h 6 h 5 h 6 h	2.2 3.8 2.6 4.22 2.3 2.5 2 2.55 1.8 3.5 2.0 1.9	25 20 0.3 47 25 32.8 — 28.8 18.4 18.5 1.6 20.34	345 420 350 440 420 359 350 347 360 387 420 362	460 620 414 505 560 448 450 440 450 503 505 437	— 10.8 5.21 12.8 — 6.119 — 5.566 — — — —	[18] [37] [38] [39] [40] [21] [41] [42] [43] [44] [27] [45]
APTES/AS AEEA/Rose bengal AEEA/Rose bengal AEEA/o-PD MPTMS/m-PD TEOS/TC APTES/AS DAMO/L-ascorbic acid APTMS/glucose APTMS/glucose	Hydrothermal Hydrothermal Hydrothermal Hydrothermal Hydrothermal Hydrothermal Stirring Stirring Stirring Stirring	180 160 160 200 180 200 RT 55 60 60	20 h 4 h 4 h 4 h 6 h 15 h 30 min 40 min 30 min 30 min	10 2.2 2.0 4.0 3.9 2.0 2 — 2.3 1.58	— 100 100 54 38.5 18 21 14.2 33 8.9	355 510 511 350 383 347 430 410 410 380	445 531 525 450 492 440 530 520 480 460	— — 4.2 4.1 8.6 3.993 10.2 — — 6.47	[46] [47] [48] [49] [50] [51] [25] [52] [53] [54]

T = reaction temperature, RT = room temperature, Ex = excitation wavelength, Em = the maximum emission wavelength, FL = photoluminescent lifetime, APTMS = (3-aminopropyl)trimethoxysilane, TC = trisodium citrate, DAMO = N-[3-(trimethoxysilyl)propyl]-ethylenediamine, PD = phenylenediamine, APTES = (3-aminopropyl)triethoxysilane, TEPA = tetraethylpentylamine, AS = ascorbate sodium, AEEA = 3-[2-(2-aminoethylamino)ethylamino]propyl-trimethoxysilane, MPTMS = 3-mercaptopropyltrimethoxysilane, TEOS = tetraethyl orthosilicate.

**Table 2:** Summary on the Performaces of SiQDs-based Biosensors.

Sample Type	Target Molecule	Excitation/Emission	Sensitivity	Dynamic Range	Reference
Tap Water	Cr_2_O_7_^2−^	362 nm/437 nm	180 nM	0.5 to 100 μM	[45]
Aqueous	Hg^2+^	347 nm/440 nm	24 nM	0.1 to 4 μM	[42]
Live Cells	pH	365 nm/440 nm	N.A.	pH 5 to pH 10	[27]
Aqueous	ClO^−^	383 nm/492 nm	13 nM	0.05 to 1.8 μM	[50]
Aqueous	DNA	359 nm/601 nm	4.3 nM	20 to 1500 nM	[68]
MCF-7 cells	MUC1	359 nm/670 nm	1.52 nM	3.33–250 nM	[75]

N.A.—Not Available.

## Data Availability

No new data were created or analyzed in this study.
